# Focus on Sex and Gender: What We Need to Know in the Management of Rheumatoid Arthritis

**DOI:** 10.3390/jpm12030499

**Published:** 2022-03-20

**Authors:** Beatrice Maranini, Alessandra Bortoluzzi, Ettore Silvagni, Marcello Govoni

**Affiliations:** Rheumatology Unit, Department of Medical Sciences, University of Ferrara, Via Luigi Borsari 46, 44121 Ferrara, Italy; alessandra.bortoluzzi@unife.it (A.B.); ettore.silvagni@edu.unife.it (E.S.); gvl@unife.it (M.G.)

**Keywords:** rheumatoid arthritis, sex, gender, treatments, clinimetrics, treat-to-target, guidelines, immune response

## Abstract

Rheumatoid arthritis (RA) is a chronic inflammatory disease, affecting mostly women with a female/male ratio of 3:1. It is characterized by symmetrical polyarthritis, leading to progressive joint damage. Sex differences have been reported in terms of disease course and characteristics, influencing patients reported outcome measures (PROMs) and pain perception, ultimately leading to male–female disparities in treatment response. Notwithstanding, sex and gender discrepancies are still under-reported in clinical trials. Therefore, there is a consistent need for a precise reference of sex and gender issues in RA studies to improve treat-to-target achievement. This narrative review explores the above-mentioned aspects of RA disease, discussing the latest core principles of RA recommendations, from safety issues to early arthritis concept and management, treat-to-target and difficult-to-treat notions, up to the most recent debate on vaccination. Our final purpose is to evaluate how sex and gender can impact current management guidelines and how this issue can be integrated for effective disease control.

## 1. Introduction

It is now well acknowledged that sex and gender of an individual are considered two distinct concepts. While the former is mainly defined by the organization of chromosomes, reproductive organs, and hormone levels, the term “gender” should be used to describe the non-physiological components of sex that are regarded as appropriate to males and females mainly from a socio-cultural point of view [1,2].

Both sex and gender can influence the appearance and development of rheumatic and autoimmune diseases. In fact, males and females are characterized, from a genetic and hormonal point of view, by a different immunological response both to foreign and self-antigens. At the same time, since gender is intimately connected to behaviors and actions, it may influence the exposure to microorganisms or access to the healthcare system [3].

Awareness of these sex- and gender-based differences has significantly contributed to understanding the differences in prevalence and incidence between males and females, not only in rheumatic and autoimmune diseases but also in cancers and infectious diseases, as well as the different responses to vaccines [1].

RA is a chronic systemic inflammatory joint disease, affecting mostly women, characterized by the presence of autoantibodies against immunoglobulin G (IgG) called rheumatoid factor and citrullinated proteins (anti-citrullinated protein antibodies). A recent study estimates the global prevalence of RA between 1980 and 2019 as 460 per 100,000 population, with variations due to geographical location and study methodology [4]. Clinically, it is characterized by symmetrical polyarthritis and extra-articular manifestations. When insufficiently treated, RA can lead to progressive joint damage and irreversible disability [5]. The current treatment strategy for RA demands a treat-to-target approach based on tight monitoring of disease activity and therapeutic adaptation when the treatment target is not achieved [6,7,8]. First-line therapy usually employs conventional synthetic DMARD as monotherapy (such as methotrexate, MTX), with a short course of glucocorticoids hopefully gradually tapered until suspension. Patients who have adverse prognostic markers or who have failed conventional synthetic DMARDs (csDMARDs) are eligible for biological DMARDs (bDMARDs) or targeted synthetic DMARDs (tsDMARDs) [6,9]. Today, despite the availability of many treatment options for patients with RA, there is little evidence helping to identify which therapy could be more effective for a particular patient at the individual level. Therefore, the current standard is a costly and time-consuming trial-and-error process of one medication after another, which may have a significant impact on the patient [10].

The role of sex and gender in the susceptibility to this disease probably involves hormonal factors and the influence of sexual dimorphism [11,12]. This sex dimorphism is less common in childhood diseases, probably because at this age, hormonal differences between males and females are negligible. On this aspect, juvenile idiopathic arthritis (JIA) is one of the few pediatric disorders where the predominance of females is more evident (F:M = 3–6.6:1) [13]. Moreover, differences regarding disease outcomes may occur. For example, chronic anterior uveitis associated with JIA affects more commonly girls, while boys may develop a more severe course [14]. The exact mechanism for this difference is far from being understood but would be quite relevant, possibly leading to a personalized therapy approach since pediatric age by a rheumatologist.

Despite the importance of these issues, many published trials and studies have not formally incorporated biological sex and gender analyses into the study design, reflecting that the progress in this area is still slow-moving [15]. The US National Institutes of Health (NIH), since 1994, has required the inclusion of women and minority groups in all NIH-funded clinical trials, together with detailed incorporation of analysis stratified by sex, gender and ethnicity in research reporting [16]. The same has been advocated by the European Medicines Agency’s International Council for Harmonisation [17]. Moreover, in 2016, the European Association of Science Editors launched their Sex and Gender Equity in Research (SAGER) guidelines, providing comprehensive guidance on reporting sex and gender differences in study design, data analysis, results, and interpretation of findings [18].

In this narrative review, we analyzed the latest literature data addressing sex/gender differences in rheumatoid arthritis (RA), focusing specifically on treatment efficacy, safety and recommendation principles of the latest RA guidelines. In order to ensure a comprehensive update on recent developments in this field is provided, search strategies were adopted, complying with recommendations for narrative reviews [19]. We searched Pubmed and Embase databases from inception up to December 2021, focusing particularly on the last 10 years of research. Sex, gender, differences, immune response, cytokine, biological therapy, immunotherapy, and their respective MESH terms were used as keywords. Specifically, we selected studies addressing biological differences between sexes in terms of inflammatory disease pathways, disease presentation picture, course and prognostic markers, drug pharmacological aspects, and therapeutic outcomes (evaluating both patient-reported outcome measures and other biomarkers). We excluded studies including other inflammatory arthropathies or arthralgia suspicious for progression to rheumatoid arthritis.

Only studies published in the English language were included, and the additional references quoted in these articles were also included. Both basic and clinical studies were selected.

## 2. Rheumatoid Arthritis Recommendations: Sex/Gender Issues

The European League Against Rheumatism (EULAR) updated the RA management recommendations in 2019 [6] to outline the latest licensed drugs and to investigate the optimal treat-to-target approach. However, several recommendations were still based on rather low levels of evidence, and many questions were raised because of variable interpretations of overarching principles based on daily clinical experience. For instance, despite the empirical evidence that males and females differ in both treatment efficacy and therapy-related adverse events [6], discrepancies persist in reporting study data between females and males, as evident in the latest of the recommendations [18,19].

Moreover, the concept of “disease modification”, which is reported in RA management guidelines, embraces not only symptoms remission and slowing of structural damage, but also improvement of physical function, quality of life, social and work capacity [6], all aspects that might be influenced by sex/gender differences.

**Figure 1:**
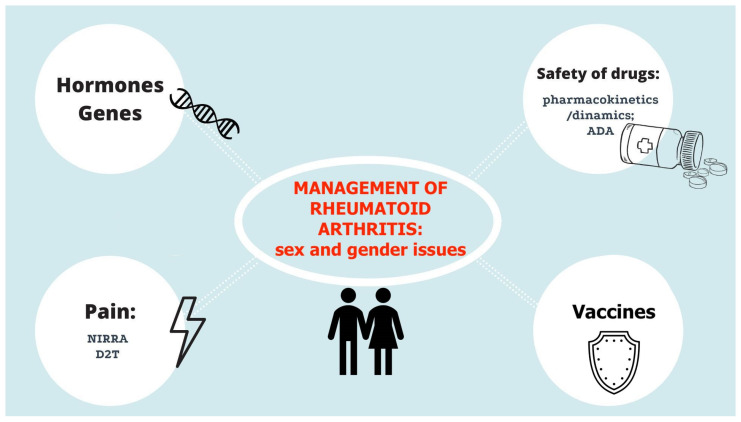
Overview of key principles of RA management from the latest sets of recommendations: both sex- and gender-based factors contribute to the illustrated aspects, affecting clinical response and outcome between females and males and, therefore, should be considered in biomedical research. *Abbreviations*: *ADA = anti-drug antibody*; *D2T = difficult-to-treat*; *NIRRA = non-inflammatory refractory RA*; *T2T = treat-to-target*.

Below, we commented about the main key points of the RA management listed in the current EULAR guidelines, according to sex and gender differences concerns. Figure 1 summarizes the main aspects of RA management recommendations focusing on sex and gender-related issues.

## 3. Safety of Drugs

EULAR has addressed safety issues on csDMARDs, bDMARDs, and tsDMARDs in RA in a series of documents over the years [20,21,22]. As widely known, sex and gender may influence drugs safety, and effectiveness in adults since pharmacokinetic/pharmacodynamic differ between women and men. Women are exposed to higher blood drug concentrations and longer drug clearance times than males, leading to female-biased adverse drug reactions [23,24]. The absence of sex-stratified pharmacokinetic information in public records raises concerns about the risks of overmedication in women. Indeed, the common practice of prescribing equal drug doses to women and men forsakes sex differences in pharmacokinetics [25].

In this paragraph, we summarized the most relevant issues about drug safety: pharmacokinetics and route of administration, with particular reference to bDMARDs, anti-drug antibodies, drug adherence, and adverse events.

Monoclonal antibodies. A review of Ternant and colleagues [26] addressed clinical pharmacokinetic and pharmacodynamic issues of monoclonal antibodies (mAbs) approved for RA treatment. Generally, the volume of distribution and clearance of mAbs increase with body size and are therefore higher in men than in women. For instance, the clearance of adalimumab and rituximab is almost 40% higher in men than women [27,28].

Overall, RA patients treated with mAbs should benefit from individualized dosing strategies, but, to the best of our knowledge, there are no studies primarily addressing sex differences in DMARDs pharmacokinetics and pharmacodynamics in the RA population. To date, probably because of the complexity of the drug pharmacokinetics, the current dosing strategy of mAbs is not based on this knowledge. With reference to the route of administration, it was hypothesized, on a speculative basis, that women, having greater subcutaneous lipid content, receive different doses of subcutaneous-administered drugs [23]. No studies addressed this issue in DMARDs-treated patients, but it may be a relevant topic since many drugs for RA are administered by subcutaneous formulation.

Anti-drug antibodies. Another relevant aspect regards the production of anti-drug antibodies (ADAs) since mAbs may elicit an immune response resulting in therapeutic failure. This risk is very high for chimeric murine mAbs; nonetheless, it is not unremarkable for fully human antibodies (e.g., adalimumab) [29,30]. ADAs can also contribute to the injection site and infusion reactions, thromboembolic events, and serum sickness, thereby raising safety concerns [31,32,33]. In a study by Hambardzumyan et al. [34], ADAs were observed more often in female RA patients treated with infliximab than in men; moreover, women were five times more likely to have undetectable serum infliximab levels. Conversely, in a recent study of Shehab et al. [35], males receiving infliximab had higher ADAs concentrations compared to females. Consistent with this, infliximab serum drug concentrations among males were lower compared to females. On the other hand, combination therapy with concurrent administration of an immunosuppressant (usually MTX) with an anti-TNF was associated with improvement in pharmacokinetics by decreasing immunogenicity and increasing serum drug concentrations, and this effect was reported to be similar between sexes. Interestingly, in this study, there were no differences in ADA and serum drug concentrations among males and females on adalimumab therapy. These conflicting results advocate the need for new targeted studies to clarify mechanisms of action of mAbs in RA and hopefully develop an appropriate design of dosing regimens.

Adherence to therapy. Adherence to biological therapies also appears to be greater in males than females, with adverse reactions being the most common reasons for therapy discontinuation [36,37]. In a meta-analysis of almost 100 studies from different countries, female sex was an independent risk factor associated with discontinuation of biologic therapies for RA [38]. Impressively, in RA patients treated with Janus kinase inhibitors (JAKis), even if the effect of JAKi on pain seems to be more relevant in males than in females, gender seems not to influence the overall clinical response, allowing men and women the same probability of reaching the therapeutic target [39].

Safety issues. About safety, the most relevant issues when using b/tsDMARDs are infections, herpes zoster (HZ) reactivation, major cardiovascular events, including venous thromboembolic events (VTE), and change in lipid levels [22]. All these factors play a key role in the choice of therapies for RA [40,41].

It was reported that men experience a greater number of adverse effects, particularly serious infection events, during biological treatments [42]. The production of cytokines, interleukins, and chemokines by innate immune cells differ between sexes, probably because of hormonal influences. Testosterone alters the T-helper 1 (Th1) response, down-regulating the production of TNF in males. However, peripheral blood mononuclear cells (PBMCs) from males present higher levels of Toll-like receptor-4 (TLR4) and TNF production following lipopolysaccharide (LPS) stimulation, compared to females’ neutrophils, which consequently results in an increased risk for septic shock in males [43].

The risk of HZ infection is increased in particular with JAKis, especially in Asian ethnicities [22,44]. Regarding Shingles, females reported a slightly increased risk compared to males both in the general population [45] and in immunocompromised patients [44,46]; moreover, the female gender appears more prone to develop post-herpetic neuralgia [46].

Again, patients with RA present a high burden of cardiovascular (CV) disease (CVD) [47]. While RA is more common in women, the 10-year risk of CVD is significantly higher in men compared to women with RA. Furthermore, males are significantly more often current/ex-smokers and display lower HDL-cholesterol and higher atherosclerosis index [48]. Nevertheless, a recent study accounted younger and female RA patients as the two subgroups with the largest underestimation of CVD risk [49]. There are putative explanations for this. It is hypothesized that due to systemic inflammation, female RA patients reach menopause earlier than healthy control, mining the evaluation of their CV risk compared to non-RA women of similar age [50,51]. Otherwise, this topic remains a controversial subject requiring further studies.

Regarding the risk of VTE, three placebo-controlled and two head-to-head trials reported a higher risk with JAKis [22]. Few studies analyzed RA cohorts to evaluate VTE risk, independently on therapies, showing an increased risk in females [52,53].

All the above-mentioned aspects should be considered in the process of treatment decision in RA to choose as much as possible the best DMARD tailored upon the individual patient.

## 4. Early Rheumatoid Arthritis and Erosive Disease

The impact of sex affecting time to reach a definite RA diagnosis was investigated only in the study of Coffey CM et al. [54]; the authors showed no significant delay in meeting 1987 and 2010 ACR/EULAR classification criteria between females and males in an administrative medical database. However, the time to meet both 1987 and 2010 criteria was slightly longer in males compared with females, and among the seronegative subgroup, females experienced a significant delay in meeting the 2010 criteria from the first clinically detected synovitis [54].

Studies that investigated sex differences in treatment response have not addressed the issue of disease duration until recent years. In a study conducted in a National Registry, Jawaheer et al. [55] explored this aspect, suggesting that in both early and established RA patients, women had similar disease activity at baseline (in terms of clinimetric indexes, physician global scores, swollen joint count, rheumatoid factor seropositivity and radiographic changes) compared to men. However, at follow-up, women developed worse disability in terms of Health Assessment Questionnaire Disability Index (HAQ-DI), larger pain visual analog scale (VAS), worse patient global assessment (PtGA), and fatigue VAS scores, and a higher tender joint count (TJC). Notably, these differences appeared to be more pronounced, although not statistically significant, in early RA. Therefore, even if males and females did not differ in terms of baseline disease characteristics, females reached significantly lower remission rates compared to men in the follow-up period. Interestingly, the aforementioned differences were observed in early RA patients but not in patients with longstanding RA (>2 years since diagnosis) [55,56], raising the question of whether early disease stages should require a different treatment stratification based on sex. Given the similar disease activity at baseline in early RA cohorts, it was postulated that the reported sex differences might only become apparent as the disease evolves [55]. The reasons and mechanisms responsible for this different sex-related behavior in early versus established RA remain elusive. Authors did not exclude that since Disease Activity Score (DAS28) scores are highly dependent on pain perception, men may have a higher threshold in reporting joint tenderness and global health in the initial stages of the disease, but as long as disease duration increases, adaptive mechanisms altering pain perception might occur, leading to more similar symptoms between men and women.

In terms of the target achievement, it was also observed that even when using the stringent criteria described by Mäkinen et al. [57] (no swollen or tender joints and normal erythrocyte sedimentation rate), women and men achieved a remission rate of 17.8% and 26.8%, respectively, after 2 years of treatment [58].

Among the poor prognostic factors, the so-called “red flags” of RA phenotypes suitable for rapid evolution, reported in the EULAR recommendations, the presence of early erosions takes a relevant space. Evidence in the literature reported controversial results. In the BARFOT (Better Anti-rheumatic Farmacotherapy) study [59], conducted among patients with early RA, erosive disease was present in 27% of men and 28% of women at the time of diagnosis. Instead, a previous study from the Mayo Clinic [60] showed opposite results. In the extensive database of Wolfe and Sharp [61], gender was not a predictor of radiographic progression. In another study by Jawaheer et al., men had significantly worse erosion, while women had worse joint space narrowing [62].

In conclusion, EULAR recommendations do not mention sex and/or gender as a negative prognostic factor, perhaps due to a low level of evidence in the current literature, in particular in terms of objective surrogates of disease activity, such as erosions, swollen joint count and autoantibodies.

## 5. Sex Interaction and Pro-Inflammatory Immune Pathways

Currently, the EULAR principles claim that patients require access to multiple drugs with a different mechanism of action to address the heterogeneity of the RA spectrum. To date, no studies addressed sex differences in multiple sequential therapies, although the production of cytokines and chemokines by innate immune cells differs between sexes [1].

Possible reasons for gender differences in RA, in fact, have been sought in sex hormones. Estrogens display a dichotomous impact on the immune system by downregulating inflammatory pathways and upregulating immunoglobulin production [63]. In healthy individuals, exposure of plasmacytoid dendritic cells (pDCs) to Toll-Like Receptor 7 (TLR7) stimulation in vitro triggers higher production of interferon-α (IFNα) in cells from women rather than from men, and this can be enhanced by 17β-oestradiol [64]. Moreover, 17β-oestradiol can show diverging effects on human-derived monocytes and macrophages: when it enhances the production of proinflammatory cytokines at low doses (e.g., IL-1, IL-6, and TNF), which contributes to increased inflammatory values, while at high concentrations it lowers the levels of these molecules [65].

Different effects of estrogens on immune function reflect not only estrogen concentrations but also the distribution and type of estrogen receptors in immune cells [1]. Higher expression of TLR7 in immune cells of females compared with males seems to cause greater cytokine production by female immune cells and is regulated by sex chromosome expression [1]. A recent study by Vasilev et al. [66] aimed to analyze serum proinflammatory profiles of female RA patients compared with healthy women to establish the relative importance of proinflammatory cytokines in different treatment options. The levels of six cytokines were determined by ELISA assays, and all of them were found significantly higher in the sera of RA females (IL6, IL17A, IL23, IL18, TNFα, IL12p40). Early RA women displayed significantly elevated IL17A levels than those with established disease; those on tocilizumab therapy showed elevated IL6 levels and decreased IL17A compared to the rest of the patients. Moreover, these data support the pivotal role of IL-18 in addition to IL6, IL17A, and TNFα as the hierarchical cytokines in the pathogenesis of RA, notably for women.

Conversely, in healthy men, testosterone is found to increase the production of anti-inflammatory cytokines, such as IL-10 [67]. At the same time, males activated CD4+ T cells have demonstrated a greater tendency to IL-17α production compared to females [68,69]. Unsurprisingly, men with androgen deficiency present higher concentrations of proinflammatory cytokines (such as IL-1, IL-2, and TNF), as well as CD4/CD8 T cell ratios [70]. Interestingly, sex hormone metabolism in RA synovial tissues may be unfavorable for females, and TNF inhibitors may alter sex hormone metabolism right at the synovial site [71].

Thus, all these findings suggest that the impact of sex on clinical response rate in RA patients is still a controversial issue and warrants further investigation. Although numerous studies have addressed sex disparities issues in RA, to date, no studies have addressed how immune therapies mechanisms of action are affected by sex and how this mediates drug efficacy. In fact, in current clinical guidelines for RA, no sex-dependent treatment regimens have ever been recommended. Consequently, considerable research in this area is worthwhile to identify whether male and female patients require different treatment approaches to ensure the best clinical response.

## 6. Treat-to-Target Approach: The Importance of Disease Activity Assessment

EULAR recommendations advocate a treat-to-target (T2T) strategy based on treatment escalation, aiming to remission or at least low disease activity [6]. Disease activity assessment is a surrogate process measure of overall RA activity, which is used to evaluate treatment response. Several metrics are available to monitor disease activity and/or functional impact, e.g., HAQ, Clinical Disease Activity Index (CDAI), and composite clinical and laboratory-based scores, such as the widely employed DAS28 score. Although validated composite disease indexes have been pivotal over the past decades to inform clinical practices, concerns regarding their subjectivity are well-acknowledged. For instance, throughout the history of HAQ, women were reported poorer scores than men [72], reasonably because women are not as physically strong as men [73]; at the same time, men did not show significant improvements in HAQ scores in studies [74]. DAS28 is heavily weighted by the tender joint count, yet objective evidence of inflammation does not necessarily correlate with patient-reported outcome measures (PROMs) such as pain [75]. Sex hormones also influence pain transmission, modulation, and perception. Testosterone increases pain threshold, whereas conflicting results were found for estrogen and progesterone [76,77]. Besides the influence of hormones, women have a greater number of pain receptors and a different expression of these receptors, for instance, considering opioid receptors [78]. Human genetic studies also revealed sex-dependent involvement of certain genes in acute and chronic pain-related traits [79]. It is also important to notice that immune cells and associated molecules, particularly T cells, were shown to display qualitative sex differences in chronic pain [79]. Pain perception differences are also highly likely affected by gender-specific ways, such as environment and social interactions, which differ between the sexes [80]. Psychosocial factors, such as expectations, stereotypes, cultural differences, pain-related beliefs, past experiences of pain, and environmental stress, seem equally important [81]. All these points could explain the overall higher pain sensitivity in women compared with men, which might elucidate the higher pain scores reported for patient questionnaires by women with RA.

A meta-analysis of Fang et al. [82] evaluated sex impact on clinical response to biological therapy in a large RA cohort of patients. Data from 5874 patients were collected, and no significant differences in ACR20 response rate were observed between women and men. However, further data analyses of ACR20 subcomponents showed high heterogeneity among studies, and therefore data should be interpreted with caution. In a recent study including RA patients treated with first-line anti-TNF therapy, the male gender was associated with higher remission/low disease activity scores after 2 years [83], and similar findings were confirmed by a recent systematic review and meta-analysis [84]. A study by Sokka et al. [42] found that women had poorer scores in all core data set measures compared to men (DAS28, SJC, American College of Rheumatology score (ACR), pain, fatigue, and depression). In the aforementioned BARFOT study, the higher DAS28 rates in women were mainly dependent on the higher pain score [59]. In a large cohort of RA patients, no significant differences in EULAR response to rituximab were recorded between men and women during the 2-years follow-up [85]. Furthermore, authors highlighted differences in remission rates according to previous anti-TNF exposure: remission rates were higher in men in the anti-TNF inadequate-responder subgroup, while in women were higher in the anti-TNF naive subgroup. In the Orencia and Rheumatoid Arthritis Registry, no differences were recorded in response to abatacept between men and women, nor in time to achieve EULAR good-or-moderate response [86]. However, DAS28, tender joint count, and global patient assessment were consistently lower in men during follow-up [86].

Data from the British Society for Rheumatology Biologics Register—RA, have shown that female gender was a negative predictor of sustained remission and low disease activity in a cohort of RA patients treated with anti-TNF drugs [87]. A worldwide observational study of Bergstra and colleagues [88] suggested, as well, that in daily practice, RA men and women present no differences in response to DMARDs treatment.

Whether or not gender is an important factor in determining treatment responses in RA is therefore currently unclear. What is known is that the persistence of RA symptoms, causing a reduction in quality of life in apparently well-controlled disease, is one of the items of the recent EULAR definition of difficult-to-treat (D2T) RA [89]. The scientific literature has recently addressed this issue, identifying two possible types of RA patients: those for whom multiple targeted therapies lack efficacy but who have persistent inflammatory pathology, which is defined as persistent inflammatory refractory RA (PIRRA), and those with supposed refractory RA who experience persistent disease activity independently from objective evidence of inflammation, which is designated as non-inflammatory refractory RA (NIRRA) [90]. Therefore, in this latter group, composite indexes should be interpreted with caution, especially if cofounders are present, such as fibromyalgia, which typically affects the female sex and enhances the pain burden hindering the achievement of the disease control [89].

## 7. Vaccination

Patients with chronic inflammatory rheumatic diseases such as RA have an increased burden of infections due to the underlying disease itself, comorbidities, and concomitant immunosuppressive treatment. In these patients, vaccines are the best preventive treatment to defend against infectious diseases.

EULAR recommendations for vaccination changed over time, from a more cautious approach in the past years to a higher and more aware vaccination referral rate in recent years [91]. However, to the best of our knowledge, no studies exist targeting sex differences in the immune response to vaccination in patients with chronic rheumatic inflammatory/autoimmune diseases.

It was acknowledged that hormonal and genetic differences between men and women might affect the safety, immunogenicity, and efficacy of vaccines [92,93]. Females usually develop higher antibody responses but may concomitantly report more adverse reactions to vaccines compared to males [92]. Because seasonal influenza vaccine is offered annually, the core body of the literature depends on such observations. In a study by Fink et al. [94] conducted on a murine model, female mice show more robust humoral and cellular immunity than male mice after the influenza vaccine. Moreover, B cells from female mice were associated with a higher expression of TLR7. Therefore, TLR7 might contribute to sex differences in vaccine efficacy [95].

Reports of local reactions, such as redness and inflammation in site injection, are consistently more frequent in females than males [96]. Moreover, even the proportion of female vaccinated reporting systemic reactions, namely joint or muscle pain, headache, back and abdominal pain, fever, chills, and hypersensitivity reactions, are consistently predominant [97,98]. Whether differences in adverse reactions among males and females reflect a gender-based reporting bias or, instead, a true sex difference has not been resolved yet [97].

The current COVID-19 pandemic also pointed out a lot of questions concerning the need for immunization in patients with inflammatory diseases and patients under immunosuppressive regimens [99]. However, the lack of sex/gender data in immunization has been a longstanding issue, and it still is, even during the COVID-19 pandemic [100]. Preliminary data from a recent meta-analysis indicate a significantly higher efficacy in the COVID-19 vaccine in men compared to women, supporting a specific sex effect on vaccination success [101]. Concerning safety, a real-world study based on the national post-marketing surveillance data for the Pfizer-BioNTech and Moderna COVID-19 vaccines in the United States found that more women reported adverse events following COVID-19 vaccination compared to males; however, men were more likely to experience serious events compared to females [102]. On the other hand, women were more prone to develop anaphylaxis reactions to the Pfizer-BioNTech vaccine in the U.S. [103], UK [104], and Japanese cohorts [105]. Sex disparity of adverse reactions was also observed following AstraZeneca Vaccine and BNT162b2 COVID-19 Vaccine in South Korea [106]. Besides, a retrospective study showed that potential thrombotic events were reported with a double risk in women following the COVID-19 AstraZeneca vaccine compared to men [107].

As previously stated, to the best of our knowledge, no studies up to now investigated sex effects on immune response following COVID-19 vaccination in the RA population. Nonetheless, female-biased adverse reactions in the general population may introduce concern about the possibility of adverse reactions and questions raised upon the possibility of RA relapse after injection [108]. It is thus imperative to consider sex and gender as central elements throughout vaccine advancement so that the lessons learned from COVID-19 vaccines will be relevant for future research and the development of other vaccines.

## 8. Conclusions

Sex and gender differences influence many aspects of RA management and should be assessed carefully during treatment choice. Unfortunately, only a few studies have addressed this topic, with many biases deriving from a female preponderance in almost all studies, confounding reported data. Furthermore, there are no current guidelines or algorithms reporting specific suggestions on sex and gender differences issues. Therefore, scientific research should increasingly embrace the need to publish sex-disaggregated data. Implementing sex and gender differences in scientific reports is not only an essential step towards equality and inclusivity but also a real endeavor to target personalized medicine, which cannot ignore different clinical responses, long-term outcomes, and adverse events observed between female and male patients. A treat-to-target management, and most of all, the treatment of the so-called difficult-to-treat rheumatoid arthritis, can no longer overlook this discussion. This attitude might help in better assessing prognosis and more precisely tailor the treatment to the individual patient.
